# Supplementary figures and images for: Conservation of transcription factor binding specificities across 600 million years of bilateria evolution (part 1 of 2)
Source: eLife. 2015 Mar 17;4:e04837. doi: 10.7554/eLife.04837 (PMC4362205; doi:10.7554/eLife.04837)

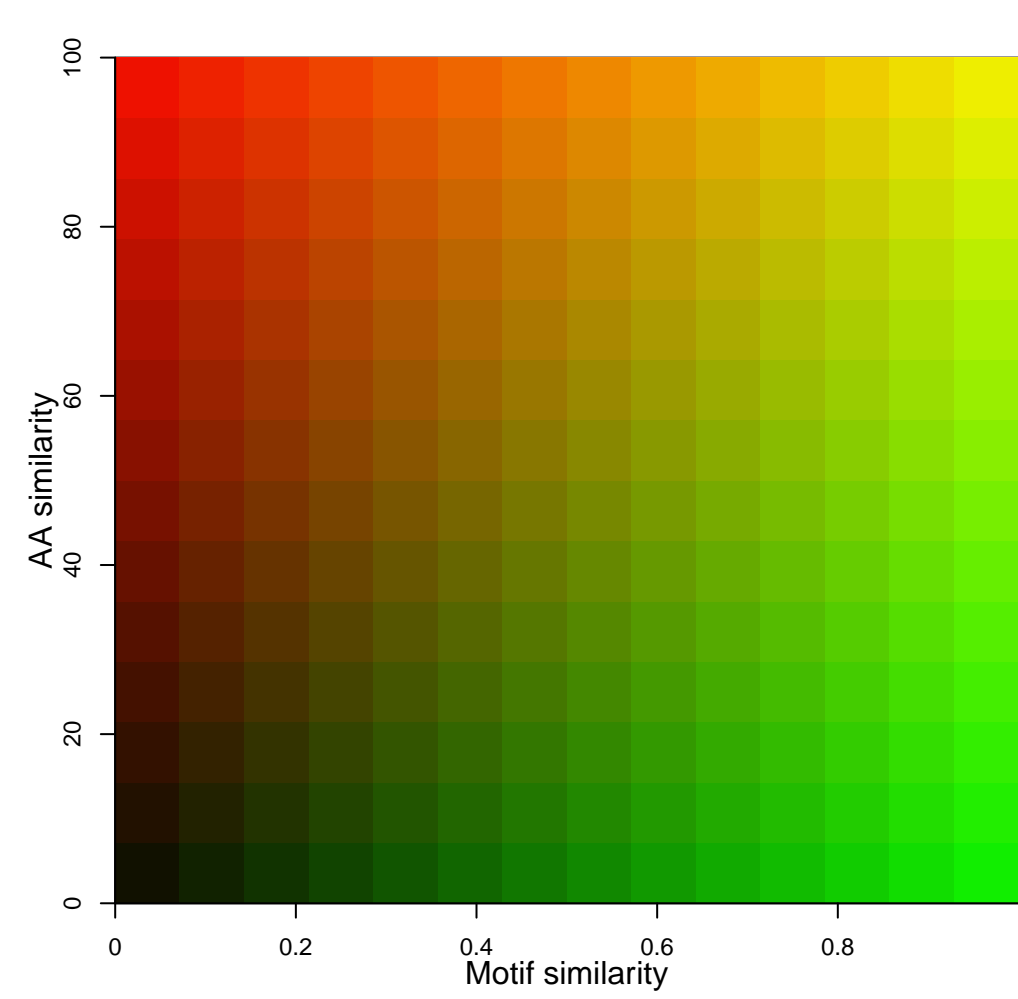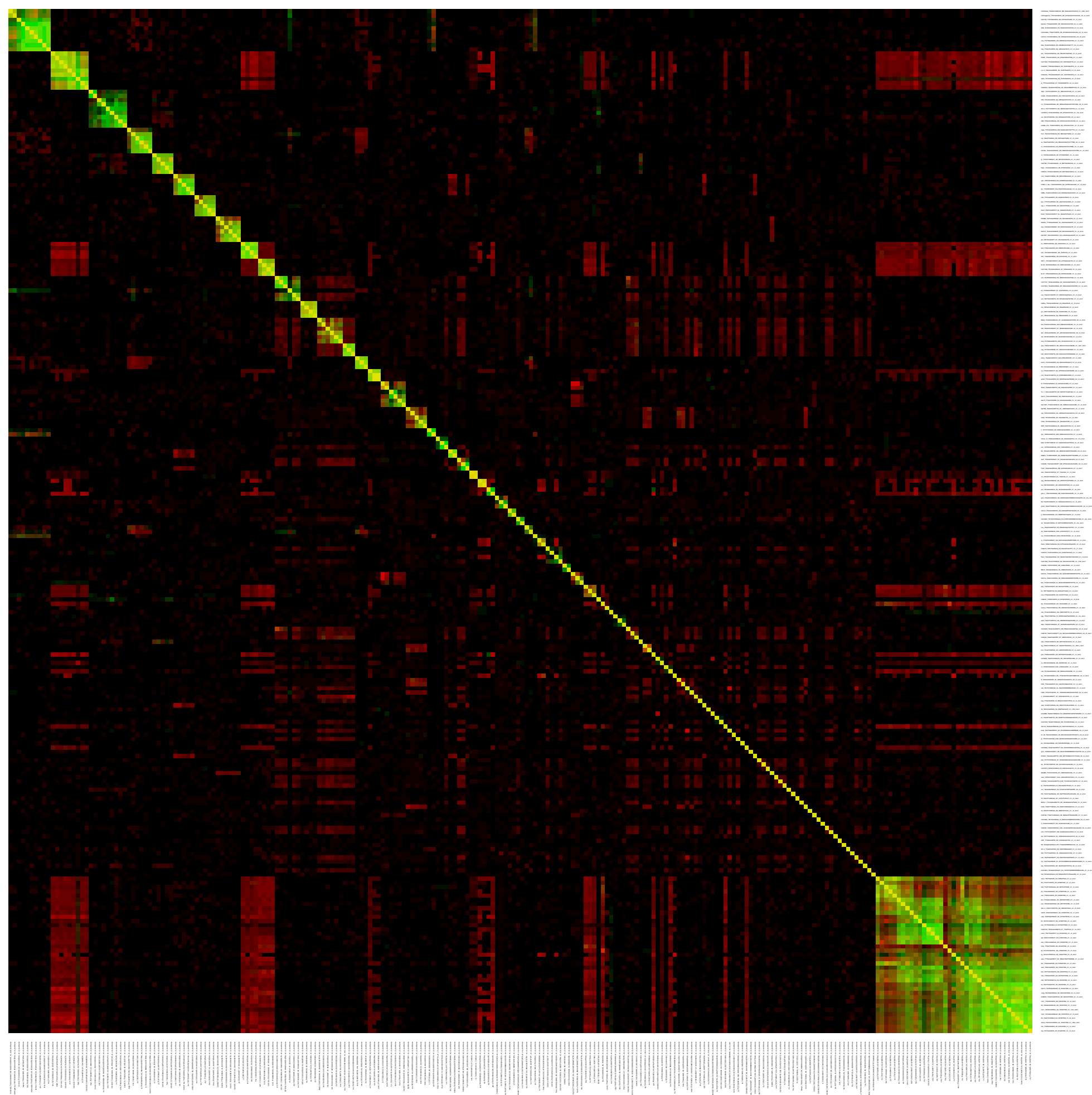

Supplement: Figure 2—source data 1. — DOI: http://dx.doi.org/10.7554/eLife.04837.019 [file elife04837s001.zip › Heatmap_fly3_6merg_represent.pdf]

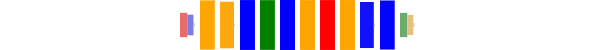

Supplement: Figure 4—source data 1. — DOI: http://dx.doi.org/10.7554/eLife.04837.027 [file elife04837s003.zip › Figure4-sourcedata1/barcode_png1/AATTC14N_U_NGGCACGTGCCN_m1_c4_short.pfm.barcode.png]

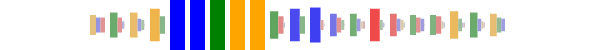

Supplement: Figure 4—source data 1. — DOI: http://dx.doi.org/10.7554/eLife.04837.027 [file elife04837s003.zip › Figure4-sourcedata1/barcode_png1/ab_SANGER_10_FBgn0259750.pfm.barcode.png]

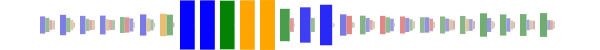

Supplement: Figure 4—source data 1. — DOI: http://dx.doi.org/10.7554/eLife.04837.027 [file elife04837s003.zip › Figure4-sourcedata1/barcode_png1/ab_SOLEXA_5_FBgn0259750.pfm.barcode.png]

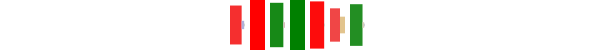

Supplement: Figure 4—source data 1. — DOI: http://dx.doi.org/10.7554/eLife.04837.027 [file elife04837s003.zip › Figure4-sourcedata1/barcode_png1/AbdA_Cell_FBgn0000014.pfm.barcode.png]

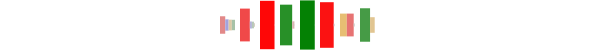

Supplement: Figure 4—source data 1. — DOI: http://dx.doi.org/10.7554/eLife.04837.027 [file elife04837s003.zip › Figure4-sourcedata1/barcode_png1/AbdA_SOLEXA_FBgn0000014.pfm.barcode.png]

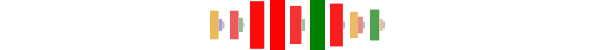

Supplement: Figure 4—source data 1. — DOI: http://dx.doi.org/10.7554/eLife.04837.027 [file elife04837s003.zip › Figure4-sourcedata1/barcode_png1/AbdB_Cell_FBgn0000015.pfm.barcode.png]

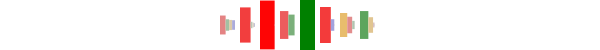

Supplement: Figure 4—source data 1. — DOI: http://dx.doi.org/10.7554/eLife.04837.027 [file elife04837s003.zip › Figure4-sourcedata1/barcode_png1/AbdB_SOLEXA_FBgn0000015.pfm.barcode.png]

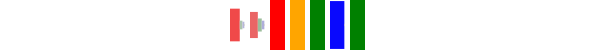

Supplement: Figure 4—source data 1. — DOI: http://dx.doi.org/10.7554/eLife.04837.027 [file elife04837s003.zip › Figure4-sourcedata1/barcode_png1/Achi_Cell_FBgn0033749.pfm.barcode.png]

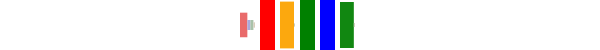

Supplement: Figure 4—source data 1. — DOI: http://dx.doi.org/10.7554/eLife.04837.027 [file elife04837s003.zip › Figure4-sourcedata1/barcode_png1/Achi_SOLEXA_FBgn0033749.pfm.barcode.png]

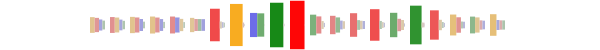

Supplement: Figure 4—source data 1. — DOI: http://dx.doi.org/10.7554/eLife.04837.027 [file elife04837s003.zip › Figure4-sourcedata1/barcode_png1/acj6_SOLEXA_5_FBgn0000028.pfm.barcode.png]

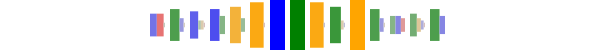

Supplement: Figure 4—source data 1. — DOI: http://dx.doi.org/10.7554/eLife.04837.027 [file elife04837s003.zip › Figure4-sourcedata1/barcode_png1/Adf1_SANGER_5_FBgn0000054.pfm.barcode.png]

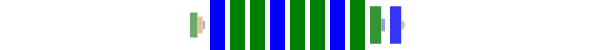

Supplement: Figure 4—source data 1. — DOI: http://dx.doi.org/10.7554/eLife.04837.027 [file elife04837s003.zip › Figure4-sourcedata1/barcode_png1/Aef1_SANGER_5_FBgn0005694.pfm.barcode.png]

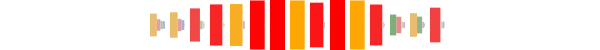

Supplement: Figure 4—source data 1. — DOI: http://dx.doi.org/10.7554/eLife.04837.027 [file elife04837s003.zip › Figure4-sourcedata1/barcode_png1/Aef1_SOLEXA_FBgn0005694.pfm.barcode.png]

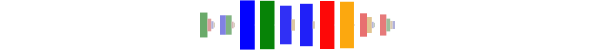

Supplement: Figure 4—source data 1. — DOI: http://dx.doi.org/10.7554/eLife.04837.027 [file elife04837s003.zip › Figure4-sourcedata1/barcode_png1/AGATA14N_U_NNCACCTGNN_m1_c4_short.pfm.barcode.png]

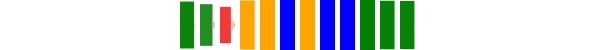

Supplement: Figure 4—source data 1. — DOI: http://dx.doi.org/10.7554/eLife.04837.027 [file elife04837s003.zip › Figure4-sourcedata1/barcode_png1/AGCAG14N_U_AATGGCGCCAAA_m1_c4_short.pfm.barcode.png]

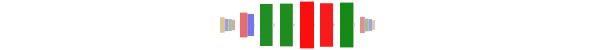

Supplement: Figure 4—source data 1. — DOI: http://dx.doi.org/10.7554/eLife.04837.027 [file elife04837s003.zip › Figure4-sourcedata1/barcode_png1/AGGGC14N_S_NYAATTAN_m1_c3_short.pfm.barcode.png]

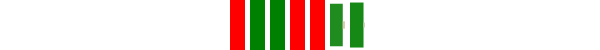

Supplement: Figure 4—source data 1. — DOI: http://dx.doi.org/10.7554/eLife.04837.027 [file elife04837s003.zip › Figure4-sourcedata1/barcode_png1/Al_Cell_FBgn0000061.pfm.barcode.png]

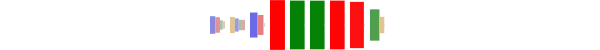

Supplement: Figure 4—source data 1. — DOI: http://dx.doi.org/10.7554/eLife.04837.027 [file elife04837s003.zip › Figure4-sourcedata1/barcode_png1/Al_SOLEXA_FBgn0000061.pfm.barcode.png]

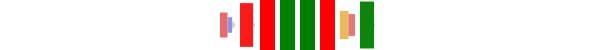

Supplement: Figure 4—source data 1. — DOI: http://dx.doi.org/10.7554/eLife.04837.027 [file elife04837s003.zip › Figure4-sourcedata1/barcode_png1/Antp_Cell_FBgn0000095.pfm.barcode.png]

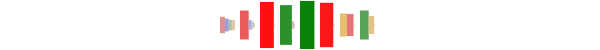

Supplement: Figure 4—source data 1. — DOI: http://dx.doi.org/10.7554/eLife.04837.027 [file elife04837s003.zip › Figure4-sourcedata1/barcode_png1/Antp_SOLEXA_FBgn0000095.pfm.barcode.png]

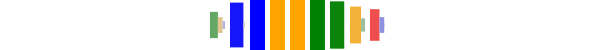

Supplement: Figure 4—source data 1. — DOI: http://dx.doi.org/10.7554/eLife.04837.027 [file elife04837s003.zip › Figure4-sourcedata1/barcode_png1/aop_SANGER_10_FBgn0000097.pfm.barcode.png]

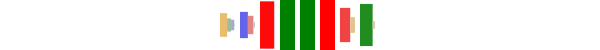

Supplement: Figure 4—source data 1. — DOI: http://dx.doi.org/10.7554/eLife.04837.027 [file elife04837s003.zip › Figure4-sourcedata1/barcode_png1/Ap_Cell_FBgn0000099.pfm.barcode.png]

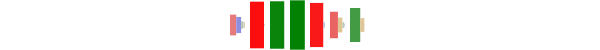

Supplement: Figure 4—source data 1. — DOI: http://dx.doi.org/10.7554/eLife.04837.027 [file elife04837s003.zip › Figure4-sourcedata1/barcode_png1/Ap_SOLEXA_FBgn0000099.pfm.barcode.png]

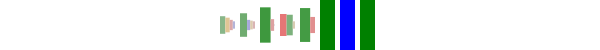

Supplement: Figure 4—source data 1. — DOI: http://dx.doi.org/10.7554/eLife.04837.027 [file elife04837s003.zip › Figure4-sourcedata1/barcode_png1/Ara_Cell_FBgn0015904.pfm.barcode.png]

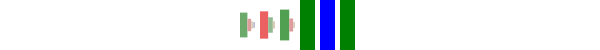

Supplement: Figure 4—source data 1. — DOI: http://dx.doi.org/10.7554/eLife.04837.027 [file elife04837s003.zip › Figure4-sourcedata1/barcode_png1/Ara_SOLEXA_FBgn0015904.pfm.barcode.png]

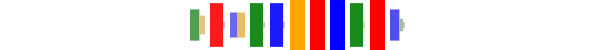

Supplement: Figure 4—source data 1. — DOI: http://dx.doi.org/10.7554/eLife.04837.027 [file elife04837s003.zip › Figure4-sourcedata1/barcode_png1/Atf-2_SANGER_5_FBgn0050420.pfm.barcode.png]

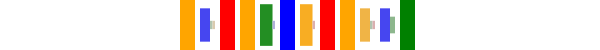

Supplement: Figure 4—source data 1. — DOI: http://dx.doi.org/10.7554/eLife.04837.027 [file elife04837s003.zip › Figure4-sourcedata1/barcode_png1/Atf6_SANGER_5_FBgn0033010.pfm.barcode.png]

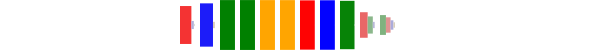

Supplement: Figure 4—source data 1. — DOI: http://dx.doi.org/10.7554/eLife.04837.027 [file elife04837s003.zip › Figure4-sourcedata1/barcode_png1/ATTAC14N_U_TCAAGGTCAWN_m1_c4_short.pfm.barcode.png]

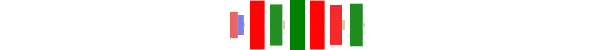

Supplement: Figure 4—source data 1. — DOI: http://dx.doi.org/10.7554/eLife.04837.027 [file elife04837s003.zip › Figure4-sourcedata1/barcode_png1/Awh_Cell_FBgn0013751.pfm.barcode.png]

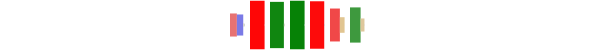

Supplement: Figure 4—source data 1. — DOI: http://dx.doi.org/10.7554/eLife.04837.027 [file elife04837s003.zip › Figure4-sourcedata1/barcode_png1/Awh_SOLEXA_FBgn0013751.pfm.barcode.png]

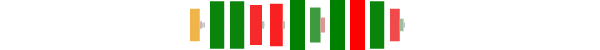

Supplement: Figure 4—source data 1. — DOI: http://dx.doi.org/10.7554/eLife.04837.027 [file elife04837s003.zip › Figure4-sourcedata1/barcode_png1/bab1_SANGER_5_FBgn0004870.pfm.barcode.png]

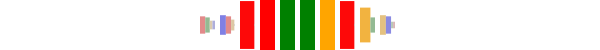

Supplement: Figure 4—source data 1. — DOI: http://dx.doi.org/10.7554/eLife.04837.027 [file elife04837s003.zip › Figure4-sourcedata1/barcode_png1/Bap_Cell_FBgn0004862.pfm.barcode.png]

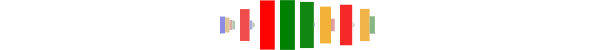

Supplement: Figure 4—source data 1. — DOI: http://dx.doi.org/10.7554/eLife.04837.027 [file elife04837s003.zip › Figure4-sourcedata1/barcode_png1/Bap_SOLEXA_FBgn0004862.pfm.barcode.png]

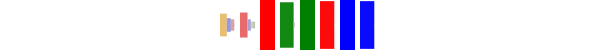

Supplement: Figure 4—source data 1. — DOI: http://dx.doi.org/10.7554/eLife.04837.027 [file elife04837s003.zip › Figure4-sourcedata1/barcode_png1/Bcd_Cell_FBgn0000166.pfm.barcode.png]

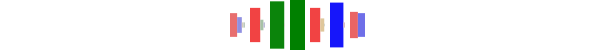

Supplement: Figure 4—source data 1. — DOI: http://dx.doi.org/10.7554/eLife.04837.027 [file elife04837s003.zip › Figure4-sourcedata1/barcode_png1/bcd_NAR_FBgn0000166.pfm.barcode.png]

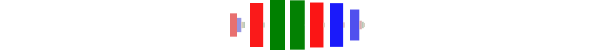

Supplement: Figure 4—source data 1. — DOI: http://dx.doi.org/10.7554/eLife.04837.027 [file elife04837s003.zip › Figure4-sourcedata1/barcode_png1/Bcd_SOLEXA_FBgn0000166.pfm.barcode.png]

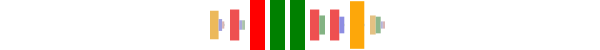

Supplement: Figure 4—source data 1. — DOI: http://dx.doi.org/10.7554/eLife.04837.027 [file elife04837s003.zip › Figure4-sourcedata1/barcode_png1/BH1_Cell_FBgn0011758.pfm.barcode.png]

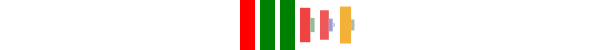

Supplement: Figure 4—source data 1. — DOI: http://dx.doi.org/10.7554/eLife.04837.027 [file elife04837s003.zip › Figure4-sourcedata1/barcode_png1/BH1_SOLEXA_FBgn0011758.pfm.barcode.png]

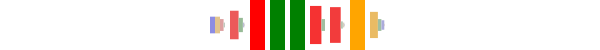

Supplement: Figure 4—source data 1. — DOI: http://dx.doi.org/10.7554/eLife.04837.027 [file elife04837s003.zip › Figure4-sourcedata1/barcode_png1/BH2_Cell_FBgn0004854.pfm.barcode.png]

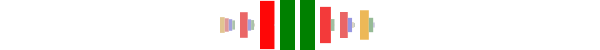

Supplement: Figure 4—source data 1. — DOI: http://dx.doi.org/10.7554/eLife.04837.027 [file elife04837s003.zip › Figure4-sourcedata1/barcode_png1/BH2_SOLEXA_FBgn0004854.pfm.barcode.png]

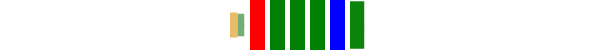

Supplement: Figure 4—source data 1. — DOI: http://dx.doi.org/10.7554/eLife.04837.027 [file elife04837s003.zip › Figure4-sourcedata1/barcode_png1/bin_SANGER_5_FBgn0045759.pfm.barcode.png]

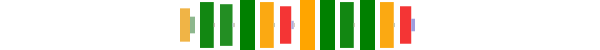

Supplement: Figure 4—source data 1. — DOI: http://dx.doi.org/10.7554/eLife.04837.027 [file elife04837s003.zip › Figure4-sourcedata1/barcode_png1/Blimp-1_NAR_FBgn0035625.pfm.barcode.png]

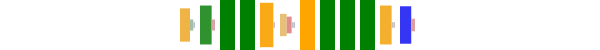

Supplement: Figure 4—source data 1. — DOI: http://dx.doi.org/10.7554/eLife.04837.027 [file elife04837s003.zip › Figure4-sourcedata1/barcode_png1/Blimp-1_SANGER_5_FBgn0035625.pfm.barcode.png]

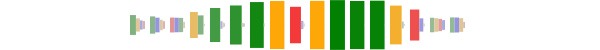

Supplement: Figure 4—source data 1. — DOI: http://dx.doi.org/10.7554/eLife.04837.027 [file elife04837s003.zip › Figure4-sourcedata1/barcode_png1/Blimp-1_SOLEXA_FBgn0035625.pfm.barcode.png]

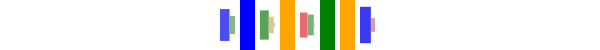

Supplement: Figure 4—source data 1. — DOI: http://dx.doi.org/10.7554/eLife.04837.027 [file elife04837s003.zip › Figure4-sourcedata1/barcode_png1/bowl_SANGER_5_FBgn0004893.pfm.barcode.png]

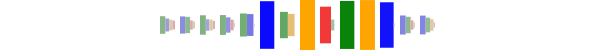

Supplement: Figure 4—source data 1. — DOI: http://dx.doi.org/10.7554/eLife.04837.027 [file elife04837s003.zip › Figure4-sourcedata1/barcode_png1/bowl_SOLEXA_FBgn0004893.pfm.barcode.png]

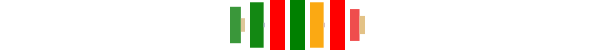

Supplement: Figure 4—source data 1. — DOI: http://dx.doi.org/10.7554/eLife.04837.027 [file elife04837s003.zip › Figure4-sourcedata1/barcode_png1/br-PA_SANGER_5_FBgn0000210.pfm.barcode.png]

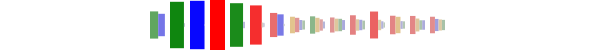

Supplement: Figure 4—source data 1. — DOI: http://dx.doi.org/10.7554/eLife.04837.027 [file elife04837s003.zip › Figure4-sourcedata1/barcode_png1/br-PA_SOLEXA_FBgn0000210.pfm.barcode.png]

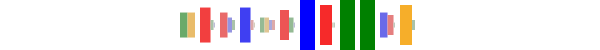

Supplement: Figure 4—source data 1. — DOI: http://dx.doi.org/10.7554/eLife.04837.027 [file elife04837s003.zip › Figure4-sourcedata1/barcode_png1/br-PE_SANGER_5_FBgn0000210.pfm.barcode.png]

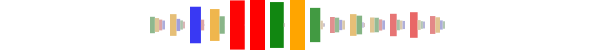

Supplement: Figure 4—source data 1. — DOI: http://dx.doi.org/10.7554/eLife.04837.027 [file elife04837s003.zip › Figure4-sourcedata1/barcode_png1/br-PE_SOLEXA_FBgn0000210.pfm.barcode.png]

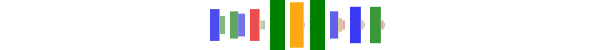

Supplement: Figure 4—source data 1. — DOI: http://dx.doi.org/10.7554/eLife.04837.027 [file elife04837s003.zip › Figure4-sourcedata1/barcode_png1/br-PL_SANGER_5_FBgn0000210.pfm.barcode.png]

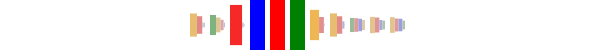

Supplement: Figure 4—source data 1. — DOI: http://dx.doi.org/10.7554/eLife.04837.027 [file elife04837s003.zip › Figure4-sourcedata1/barcode_png1/br-PL_SOLEXA_FBgn0000210.pfm.barcode.png]

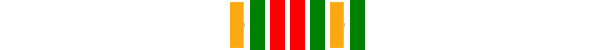

Supplement: Figure 4—source data 1. — DOI: http://dx.doi.org/10.7554/eLife.04837.027 [file elife04837s003.zip › Figure4-sourcedata1/barcode_png1/br_SANGER_10_FBgn0000210.pfm.barcode.png]

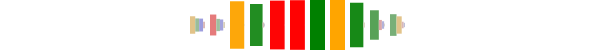

Supplement: Figure 4—source data 1. — DOI: http://dx.doi.org/10.7554/eLife.04837.027 [file elife04837s003.zip › Figure4-sourcedata1/barcode_png1/br_SOLEXA_10_FBgn0000210.pfm.barcode.png]

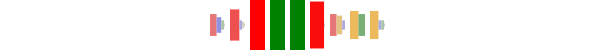

Supplement: Figure 4—source data 1. — DOI: http://dx.doi.org/10.7554/eLife.04837.027 [file elife04837s003.zip › Figure4-sourcedata1/barcode_png1/Bsh_Cell_FBgn0000529.pfm.barcode.png]

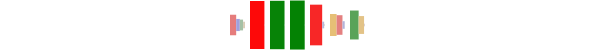

Supplement: Figure 4—source data 1. — DOI: http://dx.doi.org/10.7554/eLife.04837.027 [file elife04837s003.zip › Figure4-sourcedata1/barcode_png1/Bsh_SOLEXA_FBgn0000529.pfm.barcode.png]

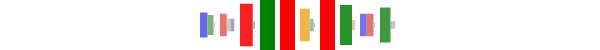

Supplement: Figure 4—source data 1. — DOI: http://dx.doi.org/10.7554/eLife.04837.027 [file elife04837s003.zip › Figure4-sourcedata1/barcode_png1/BtbVII_SANGER_5_FBgn0263108.pfm.barcode.png]

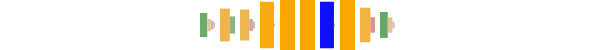

Supplement: Figure 4—source data 1. — DOI: http://dx.doi.org/10.7554/eLife.04837.027 [file elife04837s003.zip › Figure4-sourcedata1/barcode_png1/btd_NAR_FBgn0000233.pfm.barcode.png]

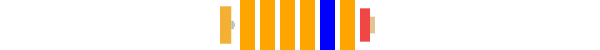

Supplement: Figure 4—source data 1. — DOI: http://dx.doi.org/10.7554/eLife.04837.027 [file elife04837s003.zip › Figure4-sourcedata1/barcode_png1/Bteb2_SANGER_2.5_FBgn0025679.pfm.barcode.png]

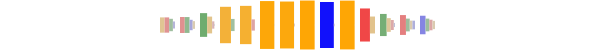

Supplement: Figure 4—source data 1. — DOI: http://dx.doi.org/10.7554/eLife.04837.027 [file elife04837s003.zip › Figure4-sourcedata1/barcode_png1/Bteb2_SOLEXA_2.5_FBgn0025679.pfm.barcode.png]

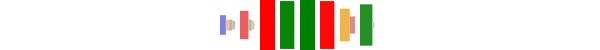

Supplement: Figure 4—source data 1. — DOI: http://dx.doi.org/10.7554/eLife.04837.027 [file elife04837s003.zip › Figure4-sourcedata1/barcode_png1/Btn_Cell_FBgn0014949.pfm.barcode.png]

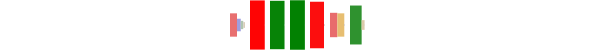

Supplement: Figure 4—source data 1. — DOI: http://dx.doi.org/10.7554/eLife.04837.027 [file elife04837s003.zip › Figure4-sourcedata1/barcode_png1/Btn_SOLEXA_FBgn0014949.pfm.barcode.png]

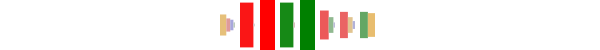

Supplement: Figure 4—source data 1. — DOI: http://dx.doi.org/10.7554/eLife.04837.027 [file elife04837s003.zip › Figure4-sourcedata1/barcode_png1/C15_Cell_FBgn0004863.pfm.barcode.png]

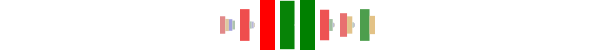

Supplement: Figure 4—source data 1. — DOI: http://dx.doi.org/10.7554/eLife.04837.027 [file elife04837s003.zip › Figure4-sourcedata1/barcode_png1/C15_SOLEXA_FBgn0004863.pfm.barcode.png]

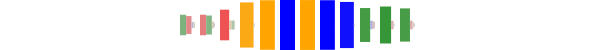

Supplement: Figure 4—source data 1. — DOI: http://dx.doi.org/10.7554/eLife.04837.027 [file elife04837s003.zip › Figure4-sourcedata1/barcode_png1/CAATT14N_U_AATGGCGCCAAA_m2_c4_short.pfm.barcode.png]

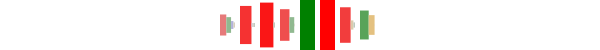

Supplement: Figure 4—source data 1. — DOI: http://dx.doi.org/10.7554/eLife.04837.027 [file elife04837s003.zip › Figure4-sourcedata1/barcode_png1/Cad_Cell_FBgn0000251.pfm.barcode.png]

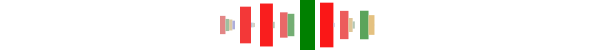

Supplement: Figure 4—source data 1. — DOI: http://dx.doi.org/10.7554/eLife.04837.027 [file elife04837s003.zip › Figure4-sourcedata1/barcode_png1/Cad_SOLEXA_FBgn0000251.pfm.barcode.png]

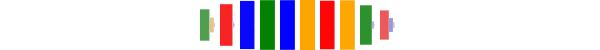

Supplement: Figure 4—source data 1. — DOI: http://dx.doi.org/10.7554/eLife.04837.027 [file elife04837s003.zip › Figure4-sourcedata1/barcode_png1/CATAT14N_S_ATCACGTGAT_m2_c4_short.pfm.barcode.png]

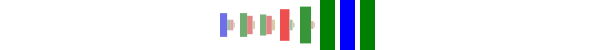

Supplement: Figure 4—source data 1. — DOI: http://dx.doi.org/10.7554/eLife.04837.027 [file elife04837s003.zip › Figure4-sourcedata1/barcode_png1/Caup_Cell_FBgn0015919.pfm.barcode.png]

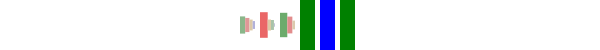

Supplement: Figure 4—source data 1. — DOI: http://dx.doi.org/10.7554/eLife.04837.027 [file elife04837s003.zip › Figure4-sourcedata1/barcode_png1/Caup_SOLEXA_FBgn0015919.pfm.barcode.png]

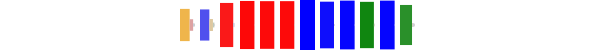

Supplement: Figure 4—source data 1. — DOI: http://dx.doi.org/10.7554/eLife.04837.027 [file elife04837s003.zip › Figure4-sourcedata1/barcode_png1/CCATA14N_V_GCTTTTCCCACA_m2_c4_short.pfm.barcode.png]

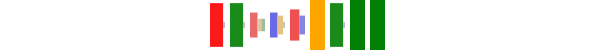

Supplement: Figure 4—source data 1. — DOI: http://dx.doi.org/10.7554/eLife.04837.027 [file elife04837s003.zip › Figure4-sourcedata1/barcode_png1/Cf2-PA_SANGER_2.5_FBgn0000286.pfm.barcode.png]

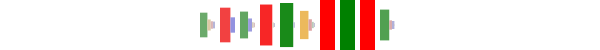

Supplement: Figure 4—source data 1. — DOI: http://dx.doi.org/10.7554/eLife.04837.027 [file elife04837s003.zip › Figure4-sourcedata1/barcode_png1/Cf2-PA_SOLEXA_FBgn0000286.pfm.barcode.png]

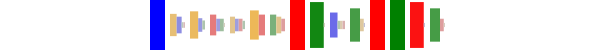

Supplement: Figure 4—source data 1. — DOI: http://dx.doi.org/10.7554/eLife.04837.027 [file elife04837s003.zip › Figure4-sourcedata1/barcode_png1/Cf2-PB_SANGER_5_FBgn0000286.pfm.barcode.png]

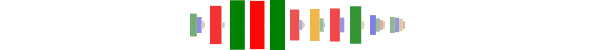

Supplement: Figure 4—source data 1. — DOI: http://dx.doi.org/10.7554/eLife.04837.027 [file elife04837s003.zip › Figure4-sourcedata1/barcode_png1/Cf2-PB_SOLEXA_FBgn0000286.pfm.barcode.png]

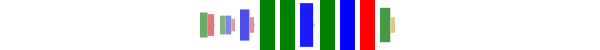

Supplement: Figure 4—source data 1. — DOI: http://dx.doi.org/10.7554/eLife.04837.027 [file elife04837s003.zip › Figure4-sourcedata1/barcode_png1/CG10267_SANGER_5_FBgn0037446.pfm.barcode.png]

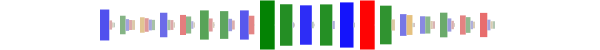

Supplement: Figure 4—source data 1. — DOI: http://dx.doi.org/10.7554/eLife.04837.027 [file elife04837s003.zip › Figure4-sourcedata1/barcode_png1/CG10267_SOLEXA_5_FBgn0037446.pfm.barcode.png]

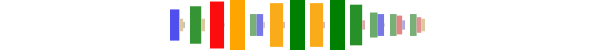

Supplement: Figure 4—source data 1. — DOI: http://dx.doi.org/10.7554/eLife.04837.027 [file elife04837s003.zip › Figure4-sourcedata1/barcode_png1/CG10904_SANGER_5_FBgn0034945.pfm.barcode.png]

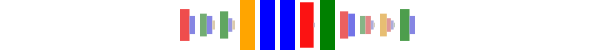

Supplement: Figure 4—source data 1. — DOI: http://dx.doi.org/10.7554/eLife.04837.027 [file elife04837s003.zip › Figure4-sourcedata1/barcode_png1/CG11071_SANGER_5_FBgn0030532.pfm.barcode.png]

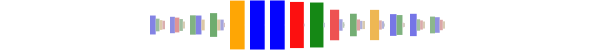

Supplement: Figure 4—source data 1. — DOI: http://dx.doi.org/10.7554/eLife.04837.027 [file elife04837s003.zip › Figure4-sourcedata1/barcode_png1/CG11071_SOLEXA_FBgn0030532.pfm.barcode.png]

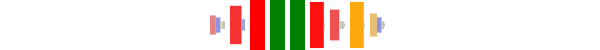

Supplement: Figure 4—source data 1. — DOI: http://dx.doi.org/10.7554/eLife.04837.027 [file elife04837s003.zip › Figure4-sourcedata1/barcode_png1/CG11085_Cell_FBgn0030408.pfm.barcode.png]

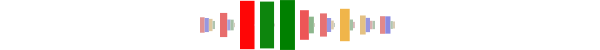

Supplement: Figure 4—source data 1. — DOI: http://dx.doi.org/10.7554/eLife.04837.027 [file elife04837s003.zip › Figure4-sourcedata1/barcode_png1/CG11085_SOLEXA_FBgn0030408.pfm.barcode.png]

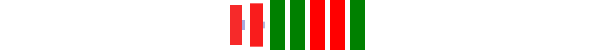

Supplement: Figure 4—source data 1. — DOI: http://dx.doi.org/10.7554/eLife.04837.027 [file elife04837s003.zip › Figure4-sourcedata1/barcode_png1/CG11294_Cell_FBgn0030058.pfm.barcode.png]

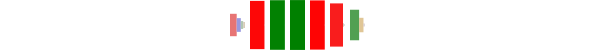

Supplement: Figure 4—source data 1. — DOI: http://dx.doi.org/10.7554/eLife.04837.027 [file elife04837s003.zip › Figure4-sourcedata1/barcode_png1/CG11294_SOLEXA_FBgn0030058.pfm.barcode.png]

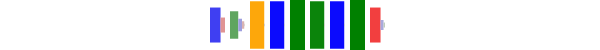

Supplement: Figure 4—source data 1. — DOI: http://dx.doi.org/10.7554/eLife.04837.027 [file elife04837s003.zip › Figure4-sourcedata1/barcode_png1/CG11504_SANGER_5_FBgn0039733.pfm.barcode.png]

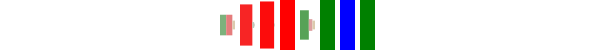

Supplement: Figure 4—source data 1. — DOI: http://dx.doi.org/10.7554/eLife.04837.027 [file elife04837s003.zip › Figure4-sourcedata1/barcode_png1/CG11617_Cell_FBgn0031232.pfm.barcode.png]

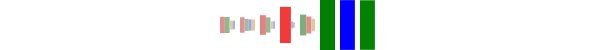

Supplement: Figure 4—source data 1. — DOI: http://dx.doi.org/10.7554/eLife.04837.027 [file elife04837s003.zip › Figure4-sourcedata1/barcode_png1/CG11617_SOLEXA_FBgn0031232.pfm.barcode.png]

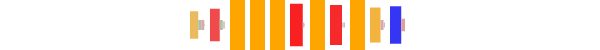

Supplement: Figure 4—source data 1. — DOI: http://dx.doi.org/10.7554/eLife.04837.027 [file elife04837s003.zip › Figure4-sourcedata1/barcode_png1/CG12029_SANGER_10_FBgn0035454.pfm.barcode.png]

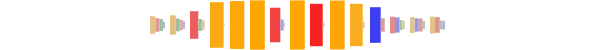

Supplement: Figure 4—source data 1. — DOI: http://dx.doi.org/10.7554/eLife.04837.027 [file elife04837s003.zip › Figure4-sourcedata1/barcode_png1/CG12029_SOLEXA_5_FBgn0035454.pfm.barcode.png]

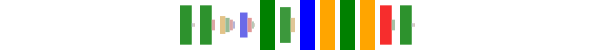

Supplement: Figure 4—source data 1. — DOI: http://dx.doi.org/10.7554/eLife.04837.027 [file elife04837s003.zip › Figure4-sourcedata1/barcode_png1/CG12155_SANGER_5_FBgn0029957.pfm.barcode.png]

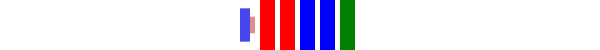

Supplement: Figure 4—source data 1. — DOI: http://dx.doi.org/10.7554/eLife.04837.027 [file elife04837s003.zip › Figure4-sourcedata1/barcode_png1/CG12236-PB_SANGER_2.5_FBgn0029822.pfm.barcode.png]

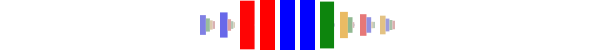

Supplement: Figure 4—source data 1. — DOI: http://dx.doi.org/10.7554/eLife.04837.027 [file elife04837s003.zip › Figure4-sourcedata1/barcode_png1/CG12236-PB_SOLEXA_FBgn0029822.pfm.barcode.png]

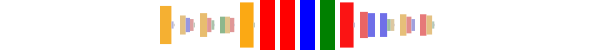

Supplement: Figure 4—source data 1. — DOI: http://dx.doi.org/10.7554/eLife.04837.027 [file elife04837s003.zip › Figure4-sourcedata1/barcode_png1/CG12236_SANGER_10_FBgn0029822.pfm.barcode.png]

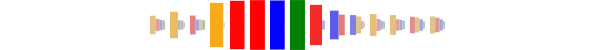

Supplement: Figure 4—source data 1. — DOI: http://dx.doi.org/10.7554/eLife.04837.027 [file elife04837s003.zip › Figure4-sourcedata1/barcode_png1/CG12236_SOLEXA_5_FBgn0029822.pfm.barcode.png]

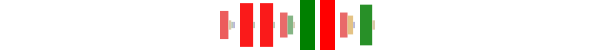

Supplement: Figure 4—source data 1. — DOI: http://dx.doi.org/10.7554/eLife.04837.027 [file elife04837s003.zip › Figure4-sourcedata1/barcode_png1/CG12361_Cell_FBgn0250756.pfm.barcode.png]

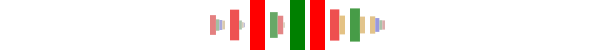

Supplement: Figure 4—source data 1. — DOI: http://dx.doi.org/10.7554/eLife.04837.027 [file elife04837s003.zip › Figure4-sourcedata1/barcode_png1/CG12361_SOLEXA_2_FBgn0250756.pfm.barcode.png]

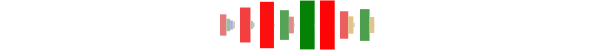

Supplement: Figure 4—source data 1. — DOI: http://dx.doi.org/10.7554/eLife.04837.027 [file elife04837s003.zip › Figure4-sourcedata1/barcode_png1/CG12361_SOLEXA_FBgn0250756.pfm.barcode.png]

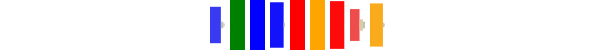

Supplement: Figure 4—source data 1. — DOI: http://dx.doi.org/10.7554/eLife.04837.027 [file elife04837s003.zip › Figure4-sourcedata1/barcode_png1/CG12605_SANGER_10_FBgn0035481.pfm.barcode.png]

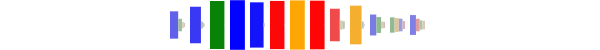

Supplement: Figure 4—source data 1. — DOI: http://dx.doi.org/10.7554/eLife.04837.027 [file elife04837s003.zip › Figure4-sourcedata1/barcode_png1/CG12605_SOLEXA_5_FBgn0035481.pfm.barcode.png]
