# Supplementary figures and images for: Conservation of transcription factor binding specificities across 600 million years of bilateria evolution (part 2 of 2)
Source: eLife. 2015 Mar 17;4:e04837. doi: 10.7554/eLife.04837 (PMC4362205; doi:10.7554/eLife.04837)

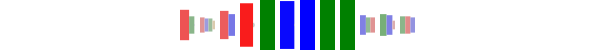

Supplement: Figure 4—source data 1. — DOI: http://dx.doi.org/10.7554/eLife.04837.027 [file elife04837s003.zip › Figure4-sourcedata1/barcode_png1/CG12768_SANGER_5_FBgn0037206.pfm.barcode.png]

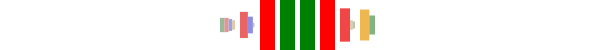

Supplement: Figure 4—source data 1. — DOI: http://dx.doi.org/10.7554/eLife.04837.027 [file elife04837s003.zip › Figure4-sourcedata1/barcode_png1/CG13424_Cell_FBgn0034520.pfm.barcode.png]

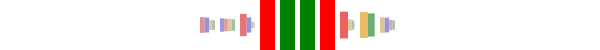

Supplement: Figure 4—source data 1. — DOI: http://dx.doi.org/10.7554/eLife.04837.027 [file elife04837s003.zip › Figure4-sourcedata1/barcode_png1/CG13424_SOLEXA_2_FBgn0034520.pfm.barcode.png]

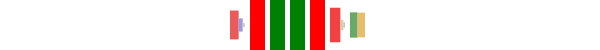

Supplement: Figure 4—source data 1. — DOI: http://dx.doi.org/10.7554/eLife.04837.027 [file elife04837s003.zip › Figure4-sourcedata1/barcode_png1/CG13424_SOLEXA_FBgn0034520.pfm.barcode.png]

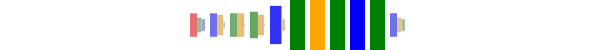

Supplement: Figure 4—source data 1. — DOI: http://dx.doi.org/10.7554/eLife.04837.027 [file elife04837s003.zip › Figure4-sourcedata1/barcode_png1/CG13897_SANGER_5_FBgn0035160.pfm.barcode.png]

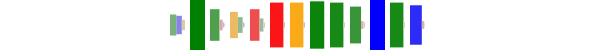

Supplement: Figure 4—source data 1. — DOI: http://dx.doi.org/10.7554/eLife.04837.027 [file elife04837s003.zip › Figure4-sourcedata1/barcode_png1/CG14962_SANGER_5_FBgn0035407.pfm.barcode.png]

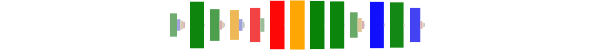

Supplement: Figure 4—source data 1. — DOI: http://dx.doi.org/10.7554/eLife.04837.027 [file elife04837s003.zip › Figure4-sourcedata1/barcode_png1/CG14962_SOLEXA_5_FBgn0035407.pfm.barcode.png]

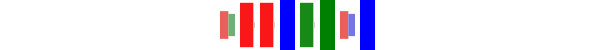

Supplement: Figure 4—source data 1. — DOI: http://dx.doi.org/10.7554/eLife.04837.027 [file elife04837s003.zip › Figure4-sourcedata1/barcode_png1/CG15601_SANGER_5_FBgn0030673.pfm.barcode.png]

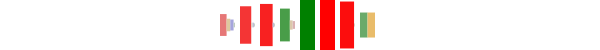

Supplement: Figure 4—source data 1. — DOI: http://dx.doi.org/10.7554/eLife.04837.027 [file elife04837s003.zip › Figure4-sourcedata1/barcode_png1/CG15696_Cell_FBgn0038833.pfm.barcode.png]

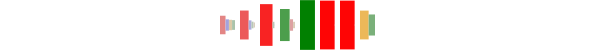

Supplement: Figure 4—source data 1. — DOI: http://dx.doi.org/10.7554/eLife.04837.027 [file elife04837s003.zip › Figure4-sourcedata1/barcode_png1/CG15696_SOLEXA_FBgn0038833.pfm.barcode.png]

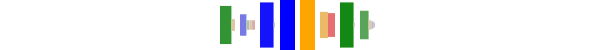

Supplement: Figure 4—source data 1. — DOI: http://dx.doi.org/10.7554/eLife.04837.027 [file elife04837s003.zip › Figure4-sourcedata1/barcode_png1/CG1621_SANGER_5_FBgn0033182.pfm.barcode.png]

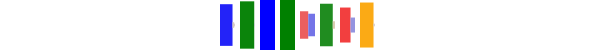

Supplement: Figure 4—source data 1. — DOI: http://dx.doi.org/10.7554/eLife.04837.027 [file elife04837s003.zip › Figure4-sourcedata1/barcode_png1/CG16778_SANGER_5_FBgn0003715.pfm.barcode.png]

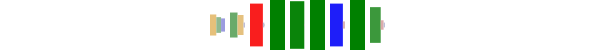

Supplement: Figure 4—source data 1. — DOI: http://dx.doi.org/10.7554/eLife.04837.027 [file elife04837s003.zip › Figure4-sourcedata1/barcode_png1/CG16899_SANGER_5_FBgn0037735.pfm.barcode.png]

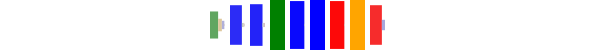

Supplement: Figure 4—source data 1. — DOI: http://dx.doi.org/10.7554/eLife.04837.027 [file elife04837s003.zip › Figure4-sourcedata1/barcode_png1/CG17181_SANGER_5_FBgn0035144.pfm.barcode.png]

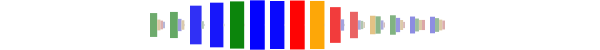

Supplement: Figure 4—source data 1. — DOI: http://dx.doi.org/10.7554/eLife.04837.027 [file elife04837s003.zip › Figure4-sourcedata1/barcode_png1/CG17181_SOLEXA_5_FBgn0035144.pfm.barcode.png]

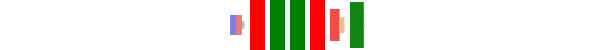

Supplement: Figure 4—source data 1. — DOI: http://dx.doi.org/10.7554/eLife.04837.027 [file elife04837s003.zip › Figure4-sourcedata1/barcode_png1/CG18599_Cell_FBgn0038592.pfm.barcode.png]

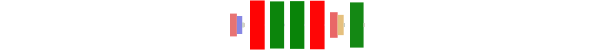

Supplement: Figure 4—source data 1. — DOI: http://dx.doi.org/10.7554/eLife.04837.027 [file elife04837s003.zip › Figure4-sourcedata1/barcode_png1/CG18599_SOLEXA_FBgn0038592.pfm.barcode.png]

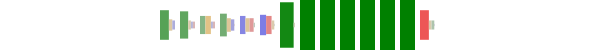

Supplement: Figure 4—source data 1. — DOI: http://dx.doi.org/10.7554/eLife.04837.027 [file elife04837s003.zip › Figure4-sourcedata1/barcode_png1/CG2052_SANGER_2.5_FBgn0039905.pfm.barcode.png]

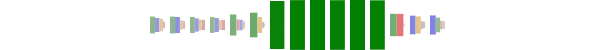

Supplement: Figure 4—source data 1. — DOI: http://dx.doi.org/10.7554/eLife.04837.027 [file elife04837s003.zip › Figure4-sourcedata1/barcode_png1/CG2052_SOLEXA_2.5_FBgn0039905.pfm.barcode.png]

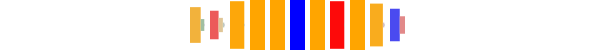

Supplement: Figure 4—source data 1. — DOI: http://dx.doi.org/10.7554/eLife.04837.027 [file elife04837s003.zip › Figure4-sourcedata1/barcode_png1/CG3065_F1-3_SANGER_2.5_FBgn0034946.pfm.barcode.png]

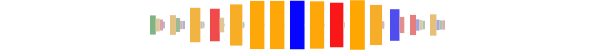

Supplement: Figure 4—source data 1. — DOI: http://dx.doi.org/10.7554/eLife.04837.027 [file elife04837s003.zip › Figure4-sourcedata1/barcode_png1/CG3065_F1-3_SOLEXA_2.5_FBgn0034946.pfm.barcode.png]

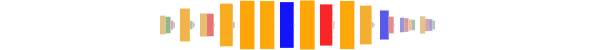

Supplement: Figure 4—source data 1. — DOI: http://dx.doi.org/10.7554/eLife.04837.027 [file elife04837s003.zip › Figure4-sourcedata1/barcode_png1/CG3065_F1-5_SOLEXA_2.5_FBgn0034946.pfm.barcode.png]

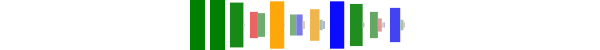

Supplement: Figure 4—source data 1. — DOI: http://dx.doi.org/10.7554/eLife.04837.027 [file elife04837s003.zip › Figure4-sourcedata1/barcode_png1/CG31670_SANGER_5_FBgn0031375.pfm.barcode.png]

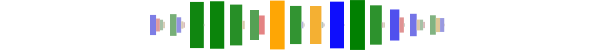

Supplement: Figure 4—source data 1. — DOI: http://dx.doi.org/10.7554/eLife.04837.027 [file elife04837s003.zip › Figure4-sourcedata1/barcode_png1/CG31670_SOLEXA_5_FBgn0031375.pfm.barcode.png]

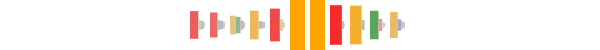

Supplement: Figure 4—source data 1. — DOI: http://dx.doi.org/10.7554/eLife.04837.027 [file elife04837s003.zip › Figure4-sourcedata1/barcode_png1/CG31782-F9-11_SOLEXA_FBgn0051782.pfm.barcode.png]

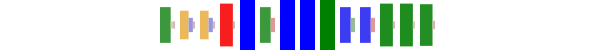

Supplement: Figure 4—source data 1. — DOI: http://dx.doi.org/10.7554/eLife.04837.027 [file elife04837s003.zip › Figure4-sourcedata1/barcode_png1/CG31782_F9-11_SANGER_5_FBgn0051782.pfm.barcode.png]

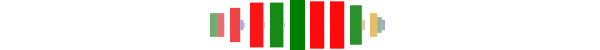

Supplement: Figure 4—source data 1. — DOI: http://dx.doi.org/10.7554/eLife.04837.027 [file elife04837s003.zip › Figure4-sourcedata1/barcode_png1/CG32105_Cell_FBgn0052105.pfm.barcode.png]

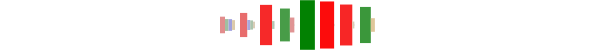

Supplement: Figure 4—source data 1. — DOI: http://dx.doi.org/10.7554/eLife.04837.027 [file elife04837s003.zip › Figure4-sourcedata1/barcode_png1/CG32105_SOLEXA_FBgn0052105.pfm.barcode.png]

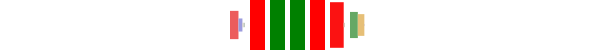

Supplement: Figure 4—source data 1. — DOI: http://dx.doi.org/10.7554/eLife.04837.027 [file elife04837s003.zip › Figure4-sourcedata1/barcode_png1/CG32532_Cell_FBgn0052532.pfm.barcode.png]

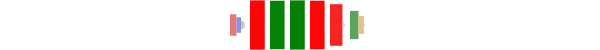

Supplement: Figure 4—source data 1. — DOI: http://dx.doi.org/10.7554/eLife.04837.027 [file elife04837s003.zip › Figure4-sourcedata1/barcode_png1/CG32532_SOLEXA_FBgn0052532.pfm.barcode.png]

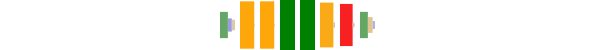

Supplement: Figure 4—source data 1. — DOI: http://dx.doi.org/10.7554/eLife.04837.027 [file elife04837s003.zip › Figure4-sourcedata1/barcode_png1/CG32830_SANGER_10_FBgn0052830.pfm.barcode.png]

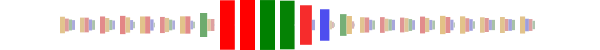

Supplement: Figure 4—source data 1. — DOI: http://dx.doi.org/10.7554/eLife.04837.027 [file elife04837s003.zip › Figure4-sourcedata1/barcode_png1/CG32830_SOLEXA_5_FBgn0052830.pfm.barcode.png]

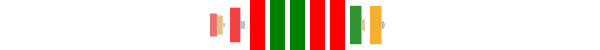

Supplement: Figure 4—source data 1. — DOI: http://dx.doi.org/10.7554/eLife.04837.027 [file elife04837s003.zip › Figure4-sourcedata1/barcode_png1/CG33980_Cell_FBgn0053980.pfm.barcode.png]

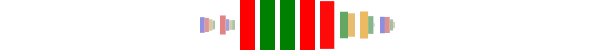

Supplement: Figure 4—source data 1. — DOI: http://dx.doi.org/10.7554/eLife.04837.027 [file elife04837s003.zip › Figure4-sourcedata1/barcode_png1/CG33980_SOLEXA_2_0_FBgn0053980.pfm.barcode.png]

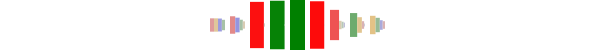

Supplement: Figure 4—source data 1. — DOI: http://dx.doi.org/10.7554/eLife.04837.027 [file elife04837s003.zip › Figure4-sourcedata1/barcode_png1/CG33980_SOLEXA_2_10_FBgn0053980.pfm.barcode.png]

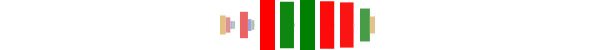

Supplement: Figure 4—source data 1. — DOI: http://dx.doi.org/10.7554/eLife.04837.027 [file elife04837s003.zip › Figure4-sourcedata1/barcode_png1/CG33980_SOLEXA_FBgn0053980.pfm.barcode.png]

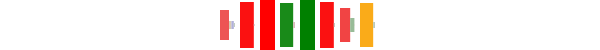

Supplement: Figure 4—source data 1. — DOI: http://dx.doi.org/10.7554/eLife.04837.027 [file elife04837s003.zip › Figure4-sourcedata1/barcode_png1/CG34031_Cell_FBgn0054031.pfm.barcode.png]

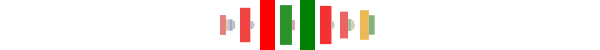

Supplement: Figure 4—source data 1. — DOI: http://dx.doi.org/10.7554/eLife.04837.027 [file elife04837s003.zip › Figure4-sourcedata1/barcode_png1/CG34031_SOLEXA_FBgn0054031.pfm.barcode.png]

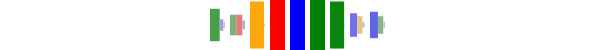

Supplement: Figure 4—source data 1. — DOI: http://dx.doi.org/10.7554/eLife.04837.027 [file elife04837s003.zip › Figure4-sourcedata1/barcode_png1/CG3407_SANGER_2.5_FBgn0031573.pfm.barcode.png]

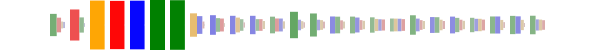

Supplement: Figure 4—source data 1. — DOI: http://dx.doi.org/10.7554/eLife.04837.027 [file elife04837s003.zip › Figure4-sourcedata1/barcode_png1/CG3407_SOLEXA_2.5_FBgn0031573.pfm.barcode.png]

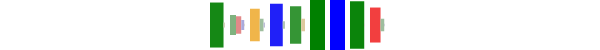

Supplement: Figure 4—source data 1. — DOI: http://dx.doi.org/10.7554/eLife.04837.027 [file elife04837s003.zip › Figure4-sourcedata1/barcode_png1/CG3838_SANGER_5_FBgn0032130.pfm.barcode.png]

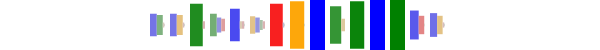

Supplement: Figure 4—source data 1. — DOI: http://dx.doi.org/10.7554/eLife.04837.027 [file elife04837s003.zip › Figure4-sourcedata1/barcode_png1/CG3919_SANGER_5_FBgn0036423.pfm.barcode.png]

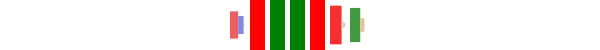

Supplement: Figure 4—source data 1. — DOI: http://dx.doi.org/10.7554/eLife.04837.027 [file elife04837s003.zip › Figure4-sourcedata1/barcode_png1/CG4136_Cell_FBgn0029775.pfm.barcode.png]

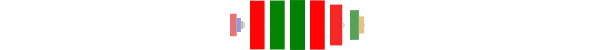

Supplement: Figure 4—source data 1. — DOI: http://dx.doi.org/10.7554/eLife.04837.027 [file elife04837s003.zip › Figure4-sourcedata1/barcode_png1/CG4136_SOLEXA_FBgn0029775.pfm.barcode.png]

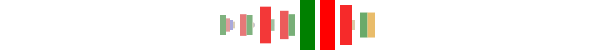

Supplement: Figure 4—source data 1. — DOI: http://dx.doi.org/10.7554/eLife.04837.027 [file elife04837s003.zip › Figure4-sourcedata1/barcode_png1/CG4328_Cell_FBgn0036274.pfm.barcode.png]

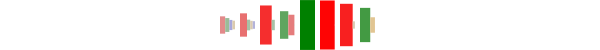

Supplement: Figure 4—source data 1. — DOI: http://dx.doi.org/10.7554/eLife.04837.027 [file elife04837s003.zip › Figure4-sourcedata1/barcode_png1/CG4328_SOLEXA_FBgn0036274.pfm.barcode.png]

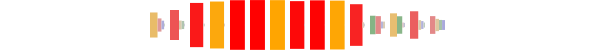

Supplement: Figure 4—source data 1. — DOI: http://dx.doi.org/10.7554/eLife.04837.027 [file elife04837s003.zip › Figure4-sourcedata1/barcode_png1/CG4360-F1-3_SOLEXA_FBgn0038787.pfm.barcode.png]

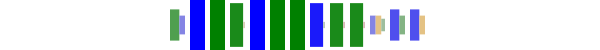

Supplement: Figure 4—source data 1. — DOI: http://dx.doi.org/10.7554/eLife.04837.027 [file elife04837s003.zip › Figure4-sourcedata1/barcode_png1/CG4360_F1-3_SANGER_2.5_FBgn0038787.pfm.barcode.png]

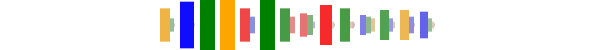

Supplement: Figure 4—source data 1. — DOI: http://dx.doi.org/10.7554/eLife.04837.027 [file elife04837s003.zip › Figure4-sourcedata1/barcode_png1/CG4404_SANGER_5_FBgn0030432.pfm.barcode.png]

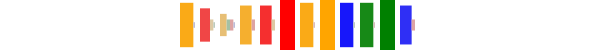

Supplement: Figure 4—source data 1. — DOI: http://dx.doi.org/10.7554/eLife.04837.027 [file elife04837s003.zip › Figure4-sourcedata1/barcode_png1/CG4854_SANGER_10_FBgn0038766.pfm.barcode.png]

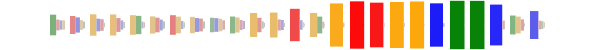

Supplement: Figure 4—source data 1. — DOI: http://dx.doi.org/10.7554/eLife.04837.027 [file elife04837s003.zip › Figure4-sourcedata1/barcode_png1/CG4854_SOLEXA_5_FBgn0038766.pfm.barcode.png]

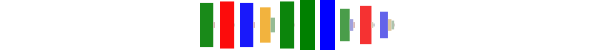

Supplement: Figure 4—source data 1. — DOI: http://dx.doi.org/10.7554/eLife.04837.027 [file elife04837s003.zip › Figure4-sourcedata1/barcode_png1/CG5180_SANGER_5_FBgn0043457.pfm.barcode.png]

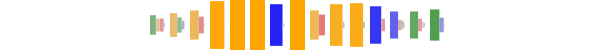

Supplement: Figure 4—source data 1. — DOI: http://dx.doi.org/10.7554/eLife.04837.027 [file elife04837s003.zip › Figure4-sourcedata1/barcode_png1/CG5669_SANGER_10_FBgn0039169.pfm.barcode.png]

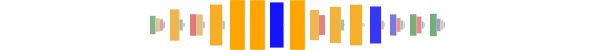

Supplement: Figure 4—source data 1. — DOI: http://dx.doi.org/10.7554/eLife.04837.027 [file elife04837s003.zip › Figure4-sourcedata1/barcode_png1/CG5669_SOLEXA_5_FBgn0039169.pfm.barcode.png]

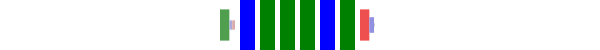

Supplement: Figure 4—source data 1. — DOI: http://dx.doi.org/10.7554/eLife.04837.027 [file elife04837s003.zip › Figure4-sourcedata1/barcode_png1/CG5953_SANGER_5_FBgn0032587.pfm.barcode.png]

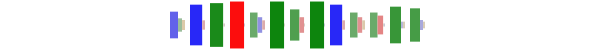

Supplement: Figure 4—source data 1. — DOI: http://dx.doi.org/10.7554/eLife.04837.027 [file elife04837s003.zip › Figure4-sourcedata1/barcode_png1/CG6276_SANGER_5_FBgn0038316.pfm.barcode.png]

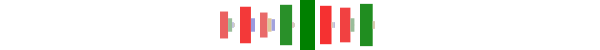

Supplement: Figure 4—source data 1. — DOI: http://dx.doi.org/10.7554/eLife.04837.027 [file elife04837s003.zip › Figure4-sourcedata1/barcode_png1/CG7056_Cell_FBgn0038852.pfm.barcode.png]

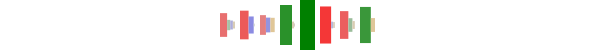

Supplement: Figure 4—source data 1. — DOI: http://dx.doi.org/10.7554/eLife.04837.027 [file elife04837s003.zip › Figure4-sourcedata1/barcode_png1/CG7056_SOLEXA_FBgn0038852.pfm.barcode.png]

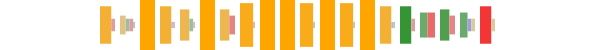

Supplement: Figure 4—source data 1. — DOI: http://dx.doi.org/10.7554/eLife.04837.027 [file elife04837s003.zip › Figure4-sourcedata1/barcode_png1/CG7368_SANGER_5_FBgn0036179.pfm.barcode.png]

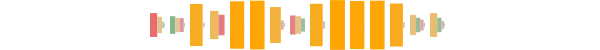

Supplement: Figure 4—source data 1. — DOI: http://dx.doi.org/10.7554/eLife.04837.027 [file elife04837s003.zip › Figure4-sourcedata1/barcode_png1/CG7368_SOLEXA_2.5_FBgn0036179.pfm.barcode.png]

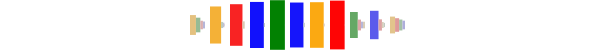

Supplement: Figure 4—source data 1. — DOI: http://dx.doi.org/10.7554/eLife.04837.027 [file elife04837s003.zip › Figure4-sourcedata1/barcode_png1/CG7386-F10-12_SOLEXA_FBgn0035691.pfm.barcode.png]

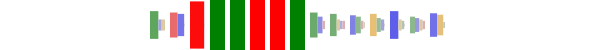

Supplement: Figure 4—source data 1. — DOI: http://dx.doi.org/10.7554/eLife.04837.027 [file elife04837s003.zip › Figure4-sourcedata1/barcode_png1/CG7745_SANGER_5_FBgn0033616.pfm.barcode.png]

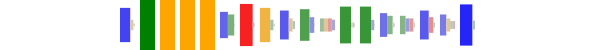

Supplement: Figure 4—source data 1. — DOI: http://dx.doi.org/10.7554/eLife.04837.027 [file elife04837s003.zip › Figure4-sourcedata1/barcode_png1/CG7928_SANGER_10_FBgn0039740.pfm.barcode.png]

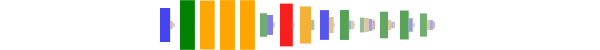

Supplement: Figure 4—source data 1. — DOI: http://dx.doi.org/10.7554/eLife.04837.027 [file elife04837s003.zip › Figure4-sourcedata1/barcode_png1/CG7928_SOLEXA_5_FBgn0039740.pfm.barcode.png]

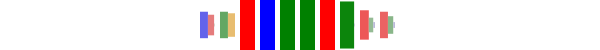

Supplement: Figure 4—source data 1. — DOI: http://dx.doi.org/10.7554/eLife.04837.027 [file elife04837s003.zip › Figure4-sourcedata1/barcode_png1/CG8281_SANGER_5_FBgn0035824.pfm.barcode.png]

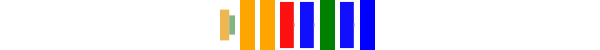

Supplement: Figure 4—source data 1. — DOI: http://dx.doi.org/10.7554/eLife.04837.027 [file elife04837s003.zip › Figure4-sourcedata1/barcode_png1/CG8319_SANGER_2.5_FBgn0037722.pfm.barcode.png]

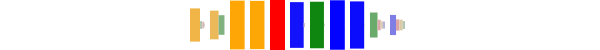

Supplement: Figure 4—source data 1. — DOI: http://dx.doi.org/10.7554/eLife.04837.027 [file elife04837s003.zip › Figure4-sourcedata1/barcode_png1/CG8319_SOLEXA_2.5_FBgn0037722.pfm.barcode.png]

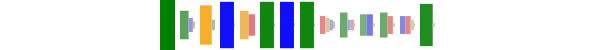

Supplement: Figure 4—source data 1. — DOI: http://dx.doi.org/10.7554/eLife.04837.027 [file elife04837s003.zip › Figure4-sourcedata1/barcode_png1/CG8765_SANGER_5_FBgn0036900.pfm.barcode.png]

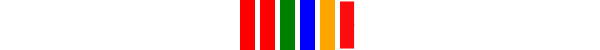

Supplement: Figure 4—source data 1. — DOI: http://dx.doi.org/10.7554/eLife.04837.027 [file elife04837s003.zip › Figure4-sourcedata1/barcode_png1/CG9437_SANGER_5_FBgn0034599.pfm.barcode.png]

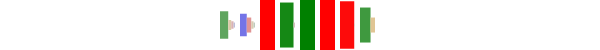

Supplement: Figure 4—source data 1. — DOI: http://dx.doi.org/10.7554/eLife.04837.027 [file elife04837s003.zip › Figure4-sourcedata1/barcode_png1/CG9876_Cell_FBgn0034821.pfm.barcode.png]

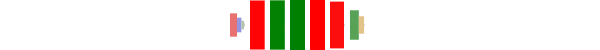

Supplement: Figure 4—source data 1. — DOI: http://dx.doi.org/10.7554/eLife.04837.027 [file elife04837s003.zip › Figure4-sourcedata1/barcode_png1/CG9876_SOLEXA_FBgn0034821.pfm.barcode.png]

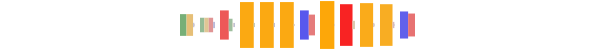

Supplement: Figure 4—source data 1. — DOI: http://dx.doi.org/10.7554/eLife.04837.027 [file elife04837s003.zip › Figure4-sourcedata1/barcode_png1/CG9895_SANGER_10_FBgn0034810.pfm.barcode.png]

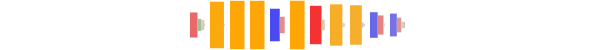

Supplement: Figure 4—source data 1. — DOI: http://dx.doi.org/10.7554/eLife.04837.027 [file elife04837s003.zip › Figure4-sourcedata1/barcode_png1/CG9895_SOLEXA_5_FBgn0034810.pfm.barcode.png]

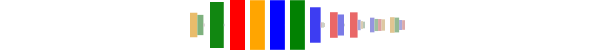

Supplement: Figure 4—source data 1. — DOI: http://dx.doi.org/10.7554/eLife.04837.027 [file elife04837s003.zip › Figure4-sourcedata1/barcode_png1/chinmo_SOLEXA_FBgn0086758.pfm.barcode.png]

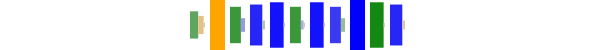

Supplement: Figure 4—source data 1. — DOI: http://dx.doi.org/10.7554/eLife.04837.027 [file elife04837s003.zip › Figure4-sourcedata1/barcode_png1/Ci_SANGER_5_FBgn0004859.pfm.barcode.png]

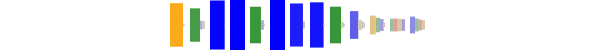

Supplement: Figure 4—source data 1. — DOI: http://dx.doi.org/10.7554/eLife.04837.027 [file elife04837s003.zip › Figure4-sourcedata1/barcode_png1/ci_SOLEXA_FBgn0004859.pfm.barcode.png]

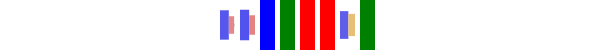

Supplement: Figure 4—source data 1. — DOI: http://dx.doi.org/10.7554/eLife.04837.027 [file elife04837s003.zip › Figure4-sourcedata1/barcode_png1/cic_SANGER_5_FBgn0028386.pfm.barcode.png]

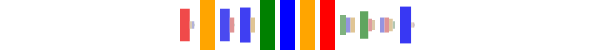

Supplement: Figure 4—source data 1. — DOI: http://dx.doi.org/10.7554/eLife.04837.027 [file elife04837s003.zip › Figure4-sourcedata1/barcode_png1/CrebA_SANGER_5_FBgn0004396.pfm.barcode.png]

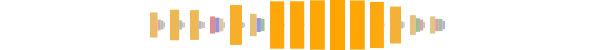

Supplement: Figure 4—source data 1. — DOI: http://dx.doi.org/10.7554/eLife.04837.027 [file elife04837s003.zip › Figure4-sourcedata1/barcode_png1/crol-F7-16_SOLEXA_FBgn0020309.pfm.barcode.png]

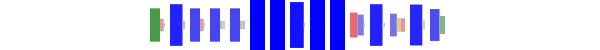

Supplement: Figure 4—source data 1. — DOI: http://dx.doi.org/10.7554/eLife.04837.027 [file elife04837s003.zip › Figure4-sourcedata1/barcode_png1/crol_F7-16_SANGER_5_FBgn0020309.pfm.barcode.png]

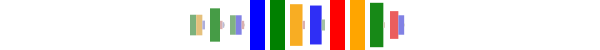

Supplement: Figure 4—source data 1. — DOI: http://dx.doi.org/10.7554/eLife.04837.027 [file elife04837s003.zip › Figure4-sourcedata1/barcode_png1/crp_SANGER_10_FBgn0001994.pfm.barcode.png]

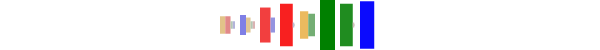

Supplement: Figure 4—source data 1. — DOI: http://dx.doi.org/10.7554/eLife.04837.027 [file elife04837s003.zip › Figure4-sourcedata1/barcode_png1/Ct_Cell_FBgn0004198.pfm.barcode.png]

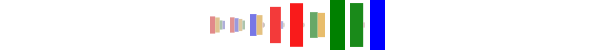

Supplement: Figure 4—source data 1. — DOI: http://dx.doi.org/10.7554/eLife.04837.027 [file elife04837s003.zip › Figure4-sourcedata1/barcode_png1/Ct_SOLEXA_FBgn0004198.pfm.barcode.png]

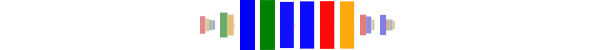

Supplement: Figure 4—source data 1. — DOI: http://dx.doi.org/10.7554/eLife.04837.027 [file elife04837s003.zip › Figure4-sourcedata1/barcode_png1/CTACA14N_U_NRCACCTGNN_m1_c4_short.pfm.barcode.png]

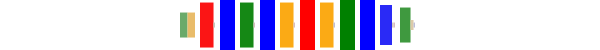

Supplement: Figure 4—source data 1. — DOI: http://dx.doi.org/10.7554/eLife.04837.027 [file elife04837s003.zip › Figure4-sourcedata1/barcode_png1/cwo_SANGER_5_FBgn0259938.pfm.barcode.png]

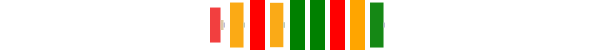

Supplement: Figure 4—source data 1. — DOI: http://dx.doi.org/10.7554/eLife.04837.027 [file elife04837s003.zip › Figure4-sourcedata1/barcode_png1/D19A-F10-12_SANGER_FBgn0022935.pfm.barcode.png]

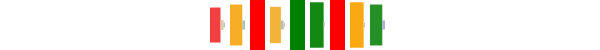

Supplement: Figure 4—source data 1. — DOI: http://dx.doi.org/10.7554/eLife.04837.027 [file elife04837s003.zip › Figure4-sourcedata1/barcode_png1/D19A_F10-12_SANGER_5_FBgn0022935.pfm.barcode.png]

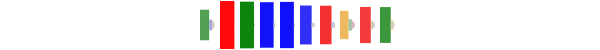

Supplement: Figure 4—source data 1. — DOI: http://dx.doi.org/10.7554/eLife.04837.027 [file elife04837s003.zip › Figure4-sourcedata1/barcode_png1/D19B-F10-12_SOLEXA_FBgn0022699.pfm.barcode.png]

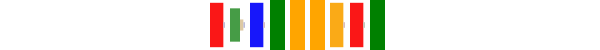

Supplement: Figure 4—source data 1. — DOI: http://dx.doi.org/10.7554/eLife.04837.027 [file elife04837s003.zip › Figure4-sourcedata1/barcode_png1/D19B_F10-12_SANGER_5_FBgn0022699.pfm.barcode.png]

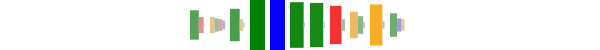

Supplement: Figure 4—source data 1. — DOI: http://dx.doi.org/10.7554/eLife.04837.027 [file elife04837s003.zip › Figure4-sourcedata1/barcode_png1/D_NAR_FBgn0000411.pfm.barcode.png]

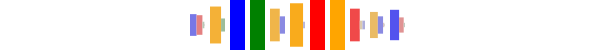

Supplement: Figure 4—source data 1. — DOI: http://dx.doi.org/10.7554/eLife.04837.027 [file elife04837s003.zip › Figure4-sourcedata1/barcode_png1/da_SANGER_10_FBgn0000413.pfm.barcode.png]

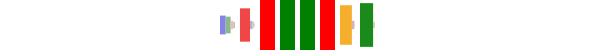

Supplement: Figure 4—source data 1. — DOI: http://dx.doi.org/10.7554/eLife.04837.027 [file elife04837s003.zip › Figure4-sourcedata1/barcode_png1/Dfd_Cell_FBgn0000439.pfm.barcode.png]

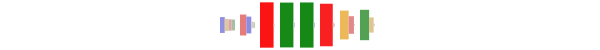

Supplement: Figure 4—source data 1. — DOI: http://dx.doi.org/10.7554/eLife.04837.027 [file elife04837s003.zip › Figure4-sourcedata1/barcode_png1/Dfd_SOLEXA_FBgn0000439.pfm.barcode.png]

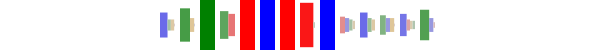

Supplement: Figure 4—source data 1. — DOI: http://dx.doi.org/10.7554/eLife.04837.027 [file elife04837s003.zip › Figure4-sourcedata1/barcode_png1/Dip3_SANGER_5_FBgn0040465.pfm.barcode.png]

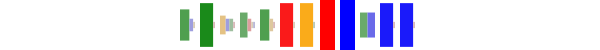

Supplement: Figure 4—source data 1. — DOI: http://dx.doi.org/10.7554/eLife.04837.027 [file elife04837s003.zip › Figure4-sourcedata1/barcode_png1/disco-r-Cl1_SANGER_5_FBgn0042650.pfm.barcode.png]

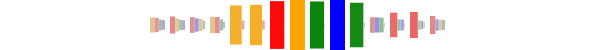

Supplement: Figure 4—source data 1. — DOI: http://dx.doi.org/10.7554/eLife.04837.027 [file elife04837s003.zip › Figure4-sourcedata1/barcode_png1/disco-r-F1-2_SOLEXA_FBgn0042650.pfm.barcode.png]

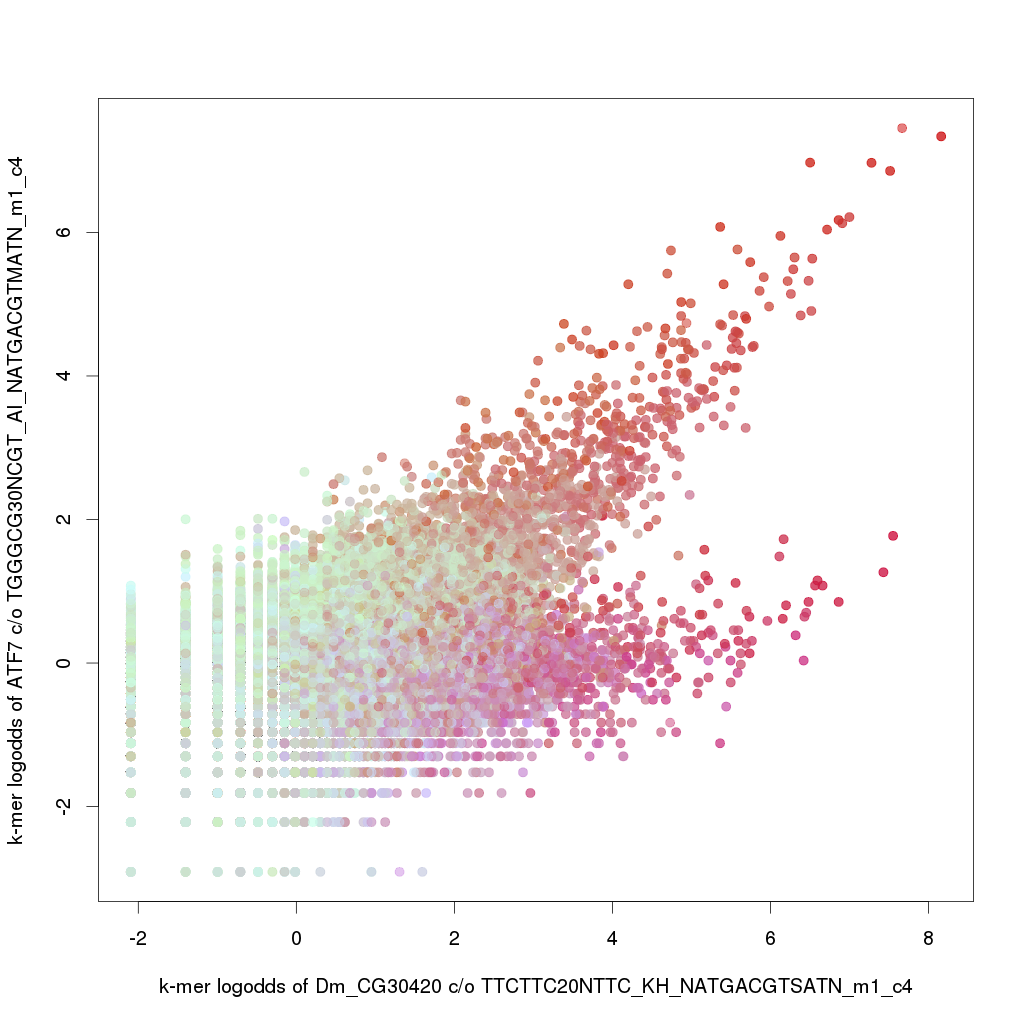

Supplement: Figure 7—source data 1. — DOI: http://dx.doi.org/10.7554/eLife.04837.031 [file elife04837s004.zip › TTCTTC20NTTC_KH_NATGACGTSATN_m1_c4_vs_TGGGCG30NCGT_AI_NATGACGTMATN_m1_c4_10mer.png]
