# Supplementary material for: Conservation of transcription factor binding specificities across 600 million years of bilateria evolution
Source: eLife. 2015 Mar 17;4:e04837. doi: 10.7554/eLife.04837 (PMC4362205; doi:10.7554/eLife.04837)

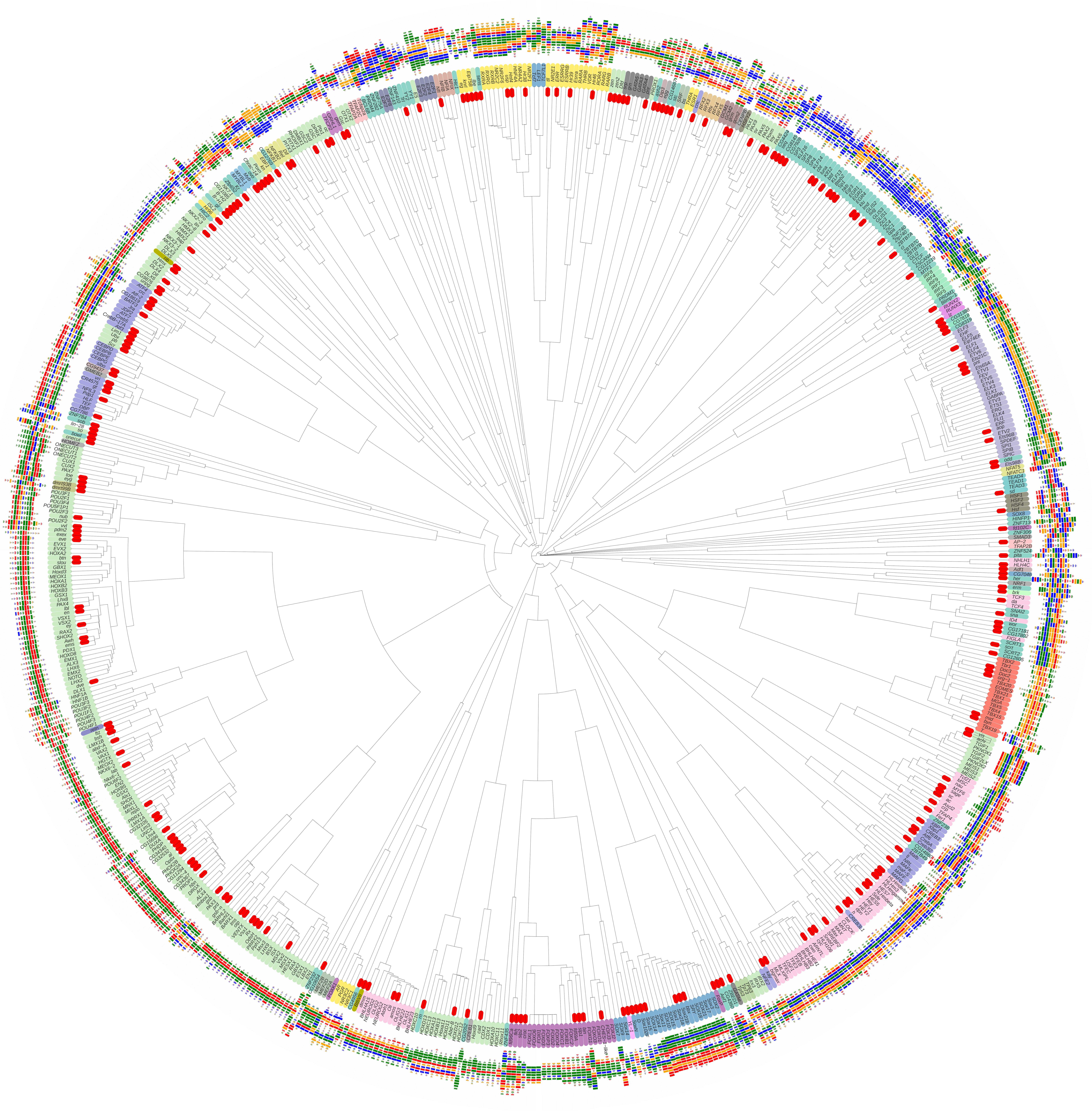

Supplement: Figure 4—source data 1. — DOI: http://dx.doi.org/10.7554/eLife.04837.027 [file All_motif_represent_dendrogram_HsV3_1_DmV3.pdf › Figure4-sourcedata1/All_motif_represent_dendrogram_HsV3_1_DmV3.pdf]
